# Lemierre's Syndrome: A Neglected Disease with Classical Features

**DOI:** 10.1155/2015/846715

**Published:** 2015-07-15

**Authors:** Andreas V. Hadjinicolaou, Yiannis Philippou

**Affiliations:** ^1^Radcliffe Department of Medicine, University of Oxford, John Radcliffe Hospital, Headington, Oxford OX3 9DU, UK; ^2^Department of Surgery, Basildon & Thurrock University Hospital, Nethermayne, Essex SS16 5NL, UK

## Abstract

We report the case of a previously healthy, immunocompetent 23-year-old male who presented to the Emergency Department with general malaise, difficulty in breathing, fever, and chest pain. He reported a two-week history of progressively worsening sore throat that he presumed to be a viral infection and thus initially neglected. However, when his condition deteriorated, he was admitted to hospital acutely unwell and in respiratory distress. He quickly developed septic shock requiring intensive care admission for inotropic support. Ultrasound and CT imaging revealed internal jugular vein thrombosis with associated septic emboli reaching the lungs to form bilateral cavitations and consequently pleural effusions. Blood cultures were positive for *Fusobacterium necrophorum*. Based on these findings, a diagnosis of Lemierre's syndrome was made. The patient was treated with appropriate antibiotics and anticoagulation and gradually recovered. He was discharged 20 days after admission with advice to complete a six-week course of antibiotics.

## 1. Introduction

Lemierre's syndrome is a condition characterised by clinical and radiological evidence of internal jugular vein (IJV) septic thrombophlebitis and bacteraemia caused primarily by the anaerobic organism* Fusobacterium necrophorum* (less frequently caused by other* Fusobacterium* species). The clinical syndrome is preceded by an oropharyngeal infection of acute onset followed by septic emboli from the original focus of infection that disseminate to the lungs.

This has been a rare illness in the era of antibiotic therapy, though it has been reported with increasing frequency in the last decade. Lemierre's syndrome should be suspected in young immunocompetent healthy patients with prolonged symptoms of pharyngitis followed by symptoms of septicaemia and pneumonia associated with respiratory distress. Lateral neck pain is also often present. Diagnosis is confirmed by identification of IJV thrombophlebitis and growth of anaerobic bacteria on blood culture. Treatment involves prolonged antibiotic therapy combined with anticoagulation.

As this syndrome is potentially fatal, recognising its clinical features early is crucial. This requires a high degree of clinical suspicion in order to perform the correct investigations promptly. Identifying the typical radiological features on US and CT imaging, along with isolating a specific family of anaerobic pathogens in the blood, is essential for diagnosis. The high prevalence of neck pain and pharyngitis as presentations in a primary care setting makes this goal even more important. Some of these cases, often attributed to viral infections or other indolent causes, could in fact represent early stages of Lemierre's disease that are misdiagnosed and consequently are only picked up very late once infection disseminates systemically and life-threatening sepsis ensues. Here we present a case of a young man with Lemierre's syndrome and review the relevant literature to illustrate key features that aid prompt diagnosis and treatment.

## 2. Case Report

### 2.1. Presentation

A previously healthy immunocompetent 23-year-old Caucasian male presented to the Emergency Department (ED) complaining of general malaise with one-week history of progressively worsening sore throat. Presuming it to be a viral infection he initially neglected the sore throat to the point that it became severe over a one-week period. Other symptoms included low-grade fever, difficulty in breathing, and inspiratory chest pain. No gastrointestinal symptoms, headache, photophobia, neck stiffness, rash, or haemoptysis were reported. The patient denied smoking, alcohol consumption, illicit drug use, or recent travel. No potential occupational hazards were identified.

On assessment in the ED the patient appeared acutely unwell with tachycardia, hypotension, hypoxaemia, and tachypnoea. Pulse was regular at 150 beats/min, blood pressure was 97/50 mmHg, respiratory rate was 28 breaths/min, temperature was 39°C, and oxygen saturation was 95% on 4 L of oxygen. On general examination there were pharyngeal erythema and cervical lymphadenopathy. Despite the presence of full range of cervical spine movements and lack of neck stiffness, neck tenderness was noted particularly on palpating the anterior triangle of the neck. Respiratory examination revealed reduced air entry with inspiratory crackles over both lung bases. Examination of the cardiovascular, abdominal, and neurological systems was unremarkable.

### 2.2. Investigations and Diagnosis

**Figure 1:**
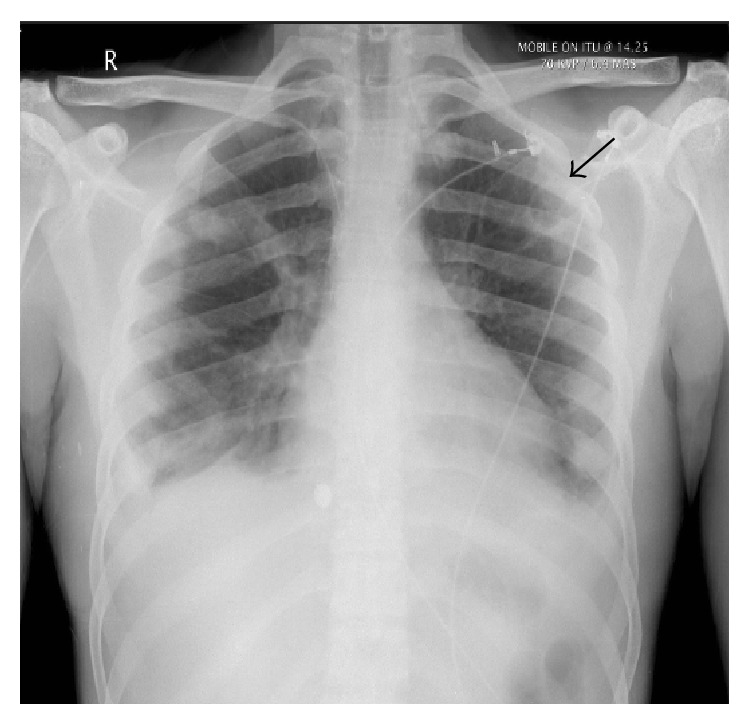
Chest X-ray on admission: arrow points to cavitating lesion in the upper zone of the left lung. Bilateral pleural effusions are evident.

Significant abnormalities on preliminary blood tests included leukocytosis (WBC: 26.4 × 10^9^/mm^3^) with extended neutrophilic shift, thrombocytopenia (platelets: 28 × 10^9^/mm^3^), raised C-reactive protein (CRP: 250 mg/L), elevated urea and creatinine (urea; 14.8 mmol/L, creatinine: 180 mmol/L), and deranged liver function tests (bilirubin: 21 mmol/L, ALP: 196 iu/L). The admission chest X-ray revealed multiple opacities throughout the lungs, a cavitating lesion within the left upper lobe, and bilateral pleural effusions (Figure 1). Urinary legionella and streptococcal test, throat swab culture, HIV, and infectious mononucleosis screens were all negative. Analysis of cerebrospinal fluid showed no abnormalities and echocardiography was also normal. The differential diagnosis at this stage included mainly infective causes such as community acquired or atypical pneumonia and tuberculosis. Disseminated intravascular coagulopathy secondary to sepsis was also considered due to the presence of thrombocytopenia and deranged coagulation profile with positive D-dimer test.

**Figure 2:**
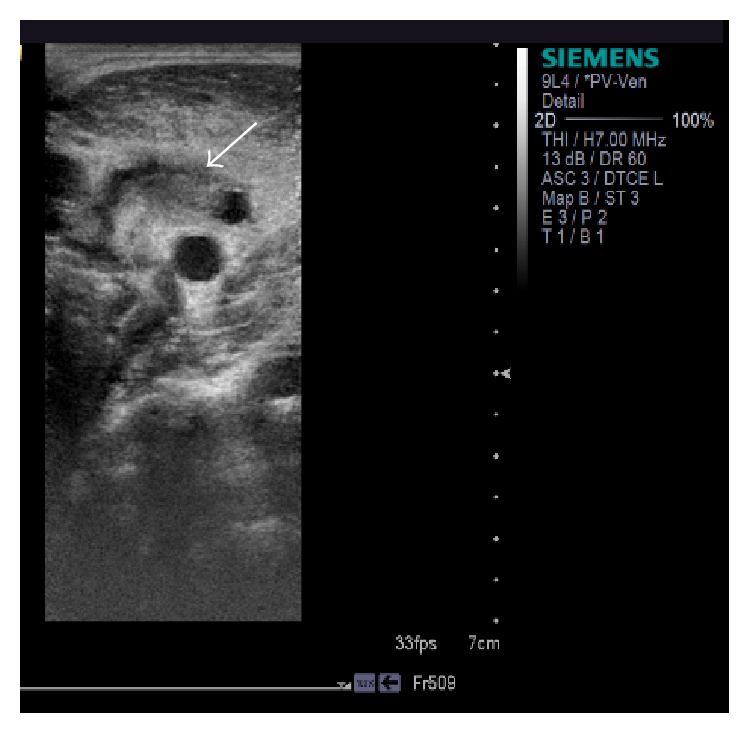
Neck ultrasound: intraluminal thrombus of the IJV (white arrow) with inability to fully compress the vein.

**Figure 3:**
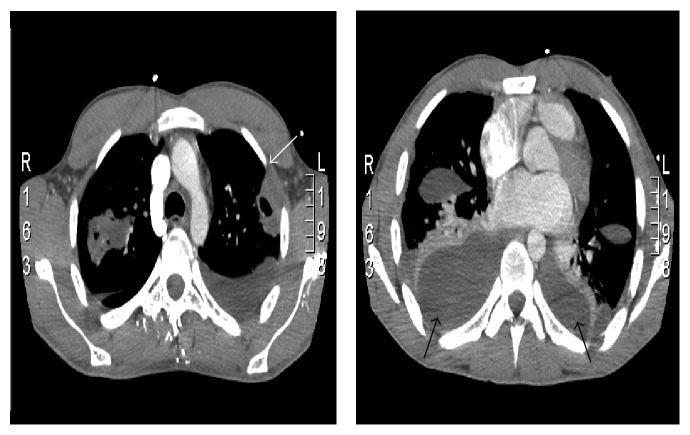
CT scan of the chest. Numerous bilateral pulmonary nodules (white arrow) can be seen with varying degrees of cavitation along with moderate bilateral pleural effusions and compressive atelectasis of both lung bases (black arrows).

As a result of persisting hypotension despite fluid resuscitation the patient was transferred to the Intensive Therapy Unit for inotropic support and continued intravenous broad-spectrum antibiotic treatment initiated on admission (benzylpenicillin and clarithromycin). Due to increasing neck tenderness further investigations using ultrasound imaging and computed tomography (CT) were initiated. Neck CT was suggestive of the presence of parapharyngeal inflammation without any signs of peritonsillar abscess. It also revealed lack of enhancement at the midportion of the right IJV and venous ultrasound confirmed IJV thrombosis (Figures 2 and 3). Chest CT revealed numerous bilateral pulmonary nodules with varying degrees of cavitation and necrosis, similar to abscesses, and pleural effusions affecting both lung bases (Figure 3).

Based on the features of sepsis observed with acute respiratory distress and chest X-ray findings severe community acquired pneumonia was initially at the top of the differentials. Tuberculosis, primary bronchogenic carcinoma, and lung metastasis were also considered. However, after CT and US scans, Lemierre's syndrome was strongly suspected.

### 2.3. Treatment and Outcome

Four days after admission, blood cultures grew Gram-negative rods that were confirmed as* Fusobacterium necrophorum* and a presumptive diagnosis of Lemierre's syndrome was made. Consequently, metronidazole was added to the existing antibiotic regimen and anticoagulation with therapeutic doses of low molecular weight heparin was initiated according to infectious disease guidelines. Following these, the patient gradually improved and was eventually discharged home 20 days after admission to complete a six-week antibiotic course.

## 3. Discussion

Lemierre's syndrome is a rare but life-threatening, bacterial-induced condition that can cause severe sepsis, acute respiratory distress syndrome, and multiorgan failure [1, 2]. It has an estimated population incidence of 2.3 per million and mainly affects young, healthy individuals [3]. Although Lemierre's syndrome is considered uncommon, recent studies suggest that the incidence of this “forgotten” disease is in fact rising [4–6]. The reason for this is believed to be the more prudent and judicious antibiotic-prescribing habits in the setting of upper respiratory tract infections (URTI) especially pharyngitis which in the past used to be treated swiftly with penicillin [7].

Lemierre's syndrome has a classic set of recognised symptoms and signs. It initially presents with a short history of a sore throat and general malaise. The initial site of infection is usually the palatine tonsils [8]. Upon examination there is pharyngeal erythema mimicking the symptoms and signs of tonsillitis or other viral URTI. Once the oropharyngeal infection is established, the causative bacteria can penetrate, either through the lymphatic system or along the fascial planes, into the adjacent blood vessels causing thrombosis and subsequent suppurative thrombophlebitis of the IJV, often forming parapharyngeal inflammation and a peritonsillar abscess along the way. Emboli from the affected IJV metastasize to the pulmonary vasculature in up to 85% of patients inflicting intense pleuritic chest pain, tachycardia, and, as septic infarcts evolve to form lung abscesses, respiratory distress [9]. For this reason chest X-ray and CT chest are useful to look for nodules, cavitations, abscesses, and pleural effusions that in this context could be formed by pulmonary septic emboli. Arthralgia, abdominal pain, diarrhea, vomiting, and, as in our case, deranged coagulation studies and abnormal liver function tests are extrapulmonary manifestations which occur due to septic emboli progressively seeding to joints and abdominal structures, including the liver, to form microabscesses [10].

*Fusobacterium necrophorum *and* Fusobacterium nucleatum *are the two bacterial species most often associated with this condition, although other bacteria have also been implicated including* Streptococcus, Bacteroides, Peptostreptococcus*, and* Eikenella* [5, 6, 11].* Fusobacteria *are nonmotile, sporulating, obligate anaerobic Gram-negative rods, which are part of the normal flora in the human upper respiratory, gastrointestinal, and female genital tracts. The exact mechanism and circumstances under which invasion and penetration of the pharyngeal mucosa occur remain to be elucidated. Current hypothesis entails the presence of a concomitant infectious agent (bacterial or viral) acting in synergy with the* Fusobacterium* to weaken host resistance and enhance virulence to cause disease [12]. This is one of the reasons that antibiotic monotherapy is not often used and a combination of agents is preferred.* Fusobacteria* have an array of virulence factors that contribute to their pathogenicity. They produce lipopolysaccharide endotoxin, hemagglutinin, leukocidin, and hemolysin. It is the presence of hemagglutinin in particular that promotes the fulminant nature of the disease by augmenting platelet aggregation and septic thrombus formation [9].

Recent reports have suggested that the occurrence of Lemierre's syndrome is more likely in individuals with certain polymorphisms in genes associated with the coagulation cascade such as Toll-like receptor 5, tissue factor 60, and Plasminogen-Activator-Inhibitor-1 genes [13]. This may suggest that the pathogenesis of this disease may depend on a complex interplay between host genetic polymorphisms in the aforementioned genes and bacterial virulence factors.

Lemierre's syndrome can be a devastating illness of young, otherwise healthy, individuals. The discovery of a thrombosed IJV on imaging is crucial to making the diagnosis. The first-line imaging of choice is ultrasound, which usually demonstrates a thrombus within the IJV causing its incompressibility and absent Doppler flow [14]. Contrast CT can subsequently confirm the presence and extent of the thrombus by displaying vessel filling defects and adjacent fat stranding [15]. The diagnosis relies on a combination of these radiological findings together with a history of pharyngotonsillitis with fever, signs of cervical lymphadenopathy with neck tenderness, and blood culture growth of an anaerobic bacterium. Aggressive antimicrobial therapy with intravenous penicillin and metronidazole, combined with surgical drainage where necessary, is the treatment of choice and has decreased the mortality rate of Lemierre's syndrome [16]. Anticoagulation often accompanies antibiotic treatment, mainly in cases with extensive thrombosis where patients are at higher risk of thromboembolic events, although there is still lack of consistent evidence for or against its routine use [17].

Although rare, we hope to raise awareness of this potentially life-threatening but treatable and often forgotten syndrome through this case. It requires a high clinical index of suspicion in order to be promptly diagnosed and treated at an early stage before fulminant sepsis occurs. Being aware of its key clinical features will aid in instigating investigations (Doppler US scan, CT scan, and blood cultures) and commencing treatment (antibiotics targeting anaerobic bacteria ± anticoagulation) in a timely fashion to provide appropriate and prompt life-saving management.

## References

[1] Lemierre A. (1936). On certain septicemias due to anaerobic organisms. *The Lancet*.

[2] Eilbert W., Singla N. (2013). Lemierre's syndrome. *International Journal of Emergency Medicine*.

[3] Hagelskjær L. H., Prag J., Malczynski J., Kristensen J. H. (1998). Incidence and clinical epidemiology of necrobacillosis, including Lemierre's syndrome, in Denmark 1990–1995. *European Journal of Clinical Microbiology and Infectious Diseases*.

[4] Karkos P. D., Asrani S., Karkos C. D. (2009). Lemierre's syndrome: a systematic review. *Laryngoscope*.

[5] Carlson E. R., Bergamo D. F., Coccia C. T. (1994). Lemierre's syndrome: two cases of a forgotten disease. *Journal of Oral and Maxillofacial Surgery*.

[6] Hoehn S., Dominguez T. E. (2002). Lemierre's syndrome: an unusual cause of sepsis and abdominal pain. *Critical Care Medicine*.

[7] Love W. E., Zaccheo M. V. (2006). Lemierre's syndrome: more judicious antibiotic prescribing habits may lead to the clinical reappearance of this often forgotten disease. *The American Journal of Medicine*.

[8] Kushawaha A., Popalzai M., El-Charabaty E., Mobarakai N. (2009). Lemierre's syndrome, reemergence of a forgotten disease: a case report. *Cases Journal*.

[9] Dorfman A., Shokoohi H., Taheri M. R. (2012). Lemierre's syndrome and rapidly deteriorating respiratory failure in the emergency department. *The American Journal of Emergency Medicine*.

[10] Stahlman G. C., DeBoer D. K., Green N. E. (1996). Fusobacterium osteomyelitis and pyarthrosis: a classic case of Lemierre's syndrome. *Journal of Pediatric Orthopaedics*.

[11] Ridgway J. M., Parikh D. A., Wright R. (2010). Lemierre syndrome: a pediatric case series and review of literature. *The American Journal of Otolaryngology—Head and Neck Medicine and Surgery*.

[12] Perrin M. A., Jankowski A., Righini C., Boubagra K., Coulomb M., Ferretti G. (2007). Imaging findings in Lemierre syndrome. *Journal de Radiologie*.

[13] Brook I., Walker R. I. (1986). The relationship between *Fusobacterium* species and other flora in mixed infection. *Journal of Medical Microbiology*.

[14] Constantin J.-M., Mira J.-P., Guerin R. (2006). Lemierre's syndrome and genetic polymorphisms: a case report. *BMC Infectious Diseases*.

[15] Screaton N. J., Ravenel J. G., Lehner P. J., Heitzman E. R., Flower C. D. R. (1999). Lemierre syndrome: forgotten but not extinct—report of four cases. *Radiology*.

[16] Riordan T., Wilson M. (2004). Lemierre's syndrome: more than a historical curiosa. *Postgraduate Medical Journal*.

[17] Phua C. K., Chadachan V. M., Acharya R. (2013). Lemierre syndrome-should we anticoagulate? A case report and review of the literature. *International Journal of Angiology*.

